# Supplementary material for: Anatomical and functional organisation of the cholinergic nervous system in the tunicate Botryllus schlosseri
Source: bioRxiv. 2026 Sep 16:2026.09.10.750344. Preprint. [Version 1] doi: 10.64898/2026.09.10.750344 (PMC13596531; doi:10.64898/2026.09.10.750344)
Supplement: Supplementary file 1 [file NIHPP2026.09.10.750344V1-supplement-1.pdf]

bioRxiv preprint doi: https://doi.org/10.64898/2026.09.10.750344; this version posted September 16, 2026. The copyright holder for this preprint (which was not certified by peer review) is the author/funder, who has granted bioRxiv a license to display the preprint in perpetuity. It is made available under a CC-BY 4.0 International license.

SUPPLEMENTARY MATERIAL

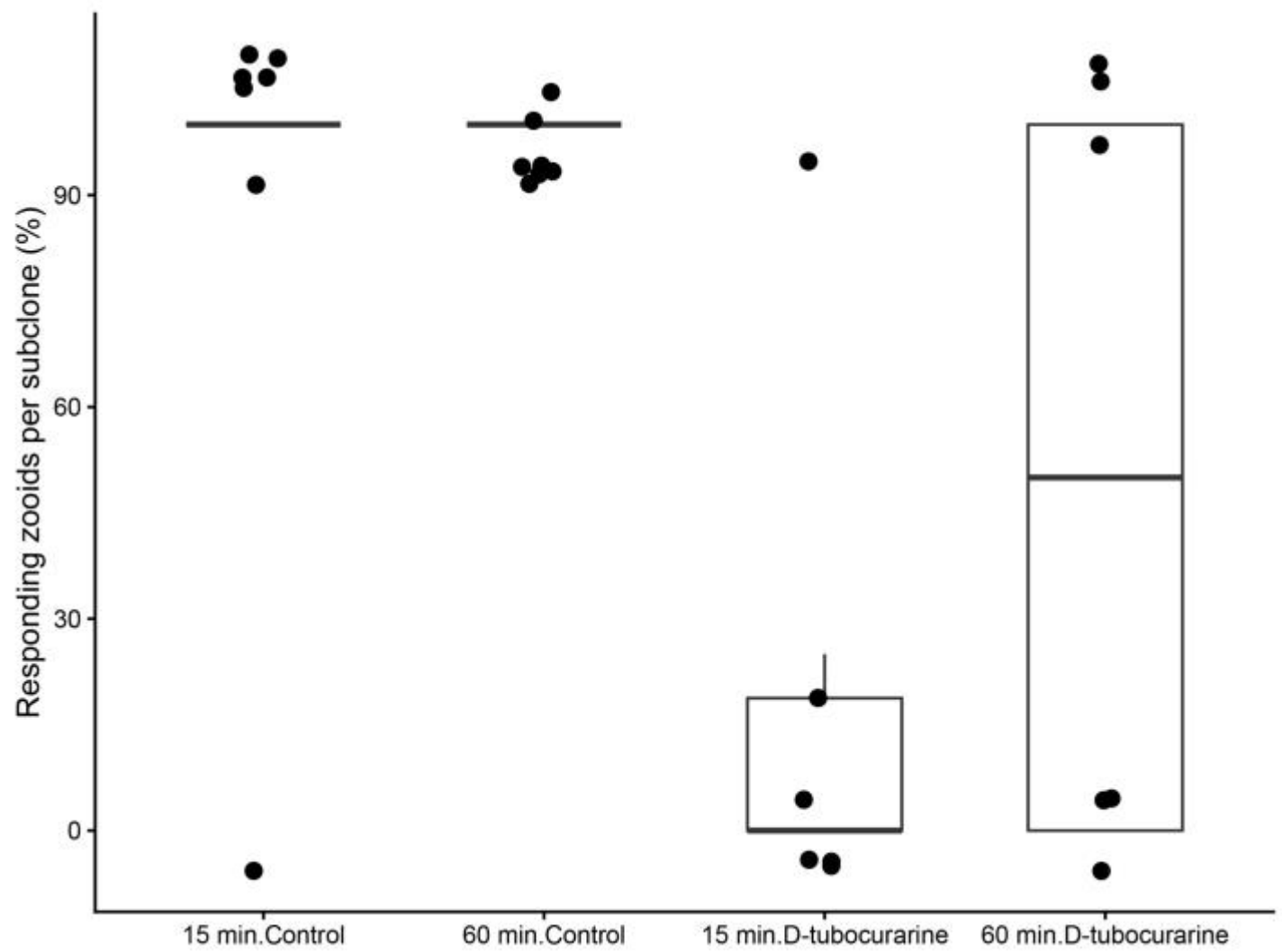

Supplementary Fig. S1. Proportion of responding zooids following focal water-jet stimulation. The fraction of zooids showing an oral siphon contraction in response to focal seawater stimulation was calculated for each control and D-tubocurarine-treated subclone at 15 and 60 min after injection. Each point represents the proportion of responding zooids within one experimental subclone/series. Zooids that did not respond to the applied stimulus were classified as non-responsive. Control subclones received filtered natural seawater, whereas treated subclones received D-tubocurarine.

Supplementary Table 1: **Raw data used for the analysis of the focal water-jet stimulation assay.** The table reports individual Stage A zooid measurements from control and D-tubocurarine-treated subclones before injection and at 15 and 60 min after injection. For each measurement, genotype, subclone/experimental-series identity, treatment condition, time point, response threshold, and response outcome are provided. `Threshold_kPa` indicates the pressure required to evoke oral siphon contraction in responding zooids. Measurements without a numerical response threshold were classified as non-responsive (`Responder = 0`) and assigned `No response` in the normalized outcome field; the original qualitative annotation is retained in the `Raw_outcome` column. Multiple zooids were sampled within each subclone at each time point, and individual zooids were not necessarily the same across time points.

Supplementary Table 2. HCR RNA-FISH probe. The table lists the gene-specific probe sets used for HCR RNA-FISH analysis.
